# Message of Acknowledgement

**DOI:** 10.1590/S2237-96222023000100032

**Published:** 2023-03-24

The editorial core group of Epidemiology and Health Services: Journal of the Brazilian National Health System (RESS) extends its thanks to the ad hoc reviewers listed below, for their collaboration and commitment in their careful evaluation of its manuscripts in 2022, which contributed greatly to the qualification, independence and ethical rigor of the journal

**Table:** 

Full Name	Institution	City	State	Country
Adeanio Almeida Lima	Secretaria Municipal de Saúde de Inhambupe	Inhambupe	BA	Brazil
Adriana Lenho de Figueiredo Pereira	Universidade do Estado do Rio de Janeiro	Rio de Janeiro	RJ	Brazil
Alanna Gomes Silva	Universidade Federal de Minas Gerais	Belo Horizonte	MG	Brazil
Albanita Gomes da Costa de Ceballos	Universidade Federal de Pernambuco	Recife	PE	Brazil
Alberto Pereira Madeiro	Universidade Federal do Piauí	Teresina	PI	Brazil
Alessandra Gaspar Sousa	Universidade de Brasília	Brasília	DF	Brazil
Alexandre San Pedro Siqueira	Instituto de Estudos em Saúde Coletiva	Rio de Janeiro	RJ	Brazil
Alícia Krüger	Organização Pan-Americana da Saúde	Brasília	DF	Brazil
Aline Almeida Bentes	Universidade Federal de Minas Gerais	Belo Horizonte	MG	Brazil
Aline Beraldo	Ministério da Saúde	Brasília	DF	Brazil
Aline Fernanda Silva Sampaio	Universidade Federal do Acre	Rio Branco	AC	Brazil
Alisson Diego Machado	Universidade de São Paulo	São Paulo	SP	Brazil
Amanda Coutinho de Souza	Organização Pan-Americana da Saúde	Brasília	DF	Brazil
Amanda Namíbia Pereira Pasklan	Universidade Federal do Maranhão	São Luís	MA	Brazil
Ana Aparecida Tessari	Universidade Federal de Santa Catarina	Florianópolis	SC	Brazil
Ana Carolina Micheletti Gomide Nogueira de Sá	Universidade Federal de Minas Gerais	Belo Horizonte	MG	Brazil
Ana Claudia Morais Godoy Figueiredo	Universidade Federal do Recôncavo da Bahia	Salvador	BA	Brazil
Ana Claudia Sierra Martin	Centro Universitário Estácio Juiz de Fora	Juiz de Fora	MG	Brazil
Ana Elisa Madalena Rinaldi	Universidade Federal de Uberlândia	Uberlândia	MG	Brazil
Ana Lúcia Sartori	Universidade Federal do Espírito Santo	Vitória	ES	Brazil
Ana Luiza de Lima Curi Hallal	Universidade Federal de Santa Catarina	Florianópolis	SC	Brazil
Ana Luiza Gomes Domingos	Universidade Federal de Viçosa	Viçosa	MG	Brazil
Ana Paula da Cunha	Escola Nacional de Saúde Pública Sergio Arouca	Manguinhos	RJ	Brazil
Ana Paula Muraro	Universidade Federal de Mato Grosso	Cuiabá	MT	Brazil
Ana Paula Rocha de Sales Miranda	Universidade Federal da Paraíba	João Pessoa	PB	Brazil
Ana Paula Sayuri Sato	Universidade Federal de Pelotas	Pelotas	RS	Brazil
André Luis Alves de Quevedo	Secretaria da Saúde do Estado do Rio Grande do Sul	Porto Alegre	RS	Brazil
André Luiz Monezi Andrade	Pontifícia Universidade Católica de Campinas	Campinas	SP	Brazil
Andrea Moreira Arrué	Universidade Federal do Paraná	Curitiba	PR	Brazil
Andressa Suelly Saturnino de Oliveira	Universidade da Integração Internacional da Lusofonia Afro-Brasileira	Fortaleza	CE	Brazil
Angelina Zanesco	Universidade Estadual Paulista ‘’Júlio de Mesquita Filho’’	Rio claro	SP	Brazil
Anna Klara Bohland	Universidade Federal de Sergipe	Aracaju	SE	Brazil
Antonia Maria da Silva Teixeira	Ministério da Saúde	Brasília	DF	Brazil
Augusto Hasiak Santo	Universidade de São Paulo	São Paulo	SP	Brazil
Barbara Almeida Soares Dias	Universidade Federal do Espírito Santo	Vitória	ES	Brazil
Barbara Reis-Santos	Rede Brasileira de Pesquisa em Tuberculose	Brasília	DF	Brazil
Bruna Cláudia Meireles Khayat	Universidade Federal do Pará	Belém	PA	Brazil
Bruna Dias Tourinho	Universidade Federal de Minas Gerais	Belo Horizonte	MG	Brazil
Bruno Gil de Carvalho Lima	Universidade Salvador	Salvador	BA	Brazil
Camila Alves Bahia	Universidade Federal de Pelotas	Pelotas	RS	Brazil
Camilo Molino Guidoni	Universidade Estadual de Londrina	Londrina	PR	Brazil
Carla Cardi Nepomuceno de Paiva	Universidade do Estado do Rio de Janeiro	Rio de Janeiro	RJ	Brazil
Carlos De Paula Silva	Universidade Federal de Minas Gerais	Belo Horizonte	MG	Brazil
Carmen Justina Gamarra	Universidade Federal da Integração Latino-Americana	Foz do Iguaçu	PR	Brazil
Carolina Maia Martins Sales	Universidade Federal do Espírito Santo	Vitória	ES	Brazil
Carolina Muller Ferreira	Universidade Estadual de Campinas	Campinas	SP	Brazil
Caroline Mary Gurgel Dias Florêncio	Universidade Federal do Ceará	Fortaleza	CE	Brazil
Cassia Barbosa Reis	Universidade Estadual de Mato Grosso do Sul	Dourados	MS	Brazil
Cássia Cristina Pinto Mendicino	Universidade Federal de Minas Gerais	Belo Horizonte	MG	Brazil
Cassio Hartmann	Instituto Federal de Alagoas	Alagoas	AL	Brazil
Celivane Cavalcanti Barbosa	Fundação Oswaldo Cruz	Recife	PE	Brazil
Christiane Soares Pereira	Instituto Nacional de Câncer	Rio de Janeiro	RJ	Brazil
Cibelle Mendes Cabral	Ministério da Saúde	Brasília	DF	Brazil
Cicero Emanoel Alves Leite	Hospital Universitário Júlio Bandeira	Cajazeiras	PB	Brazil
Cicero Luciano Alves Costa	Universidade Federal de Minas Gerais	Minas Gerais	MG	Brazil
Claudio Gruber Mann	Universidade Federal do Rio de Janeiro	Rio de Janeiro	RJ	Brazil
Cláudio Maierovitch Pessanha Henriques	Ministério da Saúde	Brasília	DF	Brazil
Cleber Tiago Cirineu	Universidad Austral de Chile	Valdivia	-	Chile
Cleonice Andrade Holanda	Universidade de Brasília	Brasília	DF	Brazil
Cleusa Marfiza Guimarães Jaccottet	Prefeitura Municipal de Pelotas	Pelotas	RS	Brazil
Cleyton Cézar Souto Silva	Faculdades de Medicina Nova Esperança	João Pessoa	PB	Brazil
Conceição Maria Oliveira	Fundação Oswaldo Cruz	Recife	PE	Brazil
Cristiano Corrêa de Azevedo Marques	Superintendência de Controle de Endemias	São Paulo	SP	Brazil
Cristine Bonfim	Fundação Joaquim Nabuco	Recife	PE	Brazil
Cynthia Andrade	Fundação Hospitalar de Minas Gerais	Belo Horizonte	MG	Brazil
Dalila Gomes	Ministério da Saúde	Brasília	DF	Brazil
Danelle da Silva Nascimento	Universidade Federal do Ceará	Fortaleza	CE	Brazil
Daniel Deivson Alves Portella	Universidade do Estado da Bahia	Salvador	BA	Brazil
Daniel Demétrio Faustino-Silva	Grupo Hospitalar Conceição	Rio Grande do Sul	RS	Brazil
Daniela Cristina Moreira Figueiredo	Universidade de Brasília	Brasília	DF	Brazil
Daniela de Almeida Pereira Duarte	Universidade Federal de Viçosa	Viçosa	MG	Brazil
Daniela Ribeiro Castilho	Universidade Federal do Pará	Belém	PA	Brazil
Daniele Andreza da Cruz Ferreira	Universidade Federal de Minas Gerais	Belo Horizonte	MG	Brazil
Danielle Teles da Cruz	Universidade Federal de Juiz de Fora	Juiz de Fora	MG	Brazil
Danielly Batista Xavier	Universidade de Brasília	Brasília	DF	Brazil
Danilo Marcelo Araujo dos Santos	Universidade Federal do Maranhão	São Luís	MA	Brazil
Danúbia da Cunha Saraiva	Instituto Nacional de Câncer	Rio de Janeiro	RJ	Brazil
Danúbia Hillesheim	Universidade Federal de Santa Catarina	Florianópolis	SC	Brazil
Daphne Rattner	Universidade de Brasília	Brasília	DF	Brazil
Debora Castanheira	Fundação Oswaldo Cruz	Rio de Janeiro	RJ	Brazil
Débora Souto-Souza	Universidade Federal dos Vales do Jequitinhonha e Mucuri	Diamantina	MG	Brazil
Edison Vieira de Melo Junior	Ministério da Saúde	Brasília	DF	Brazil
Edla Carvalho Lima Porto	Universidade Estadual de Feira de Santana	Feira de Santana	BA	Brazil
Edson Zangiacomi Martinez	Universidade de São Paulo	São Paulo	SP	Brazil
Elaine Cristina Navarro	Faculdade Eduvale de Avaré	Avaré	SP	Brazil
Elaine Tomasi	Universidade Federal de Pelotas	Pelotas	RS	Brazil
Eliana Carolina Vespero	Universidade Estadual de Londrina	Londrina	PR	Brazil
Eliane Rolim Holanda	Universidade Federal de Pernambuco	Vitória de Santo Antão	PE	Brazil
Eliete Albano de Azevedo Guimarães	Universidade Federal de São João del-Rei	São João del-Rei	MG	Brazil
Emanuele Cordeiro Chaves	Universidade Federal de Uberlândia	Uberlândia	MG	Brazil
Emília Vitoria	Universidade de Brasília	Brasília	DF	Brazil
Eunaihara Ligia Lira Marques	Instituto de Assistência Médica ao Servidor Público Estadual	São Paulo	SP	Brazil
Fabia Alexandra Pottes Alves	Universidade Federal de Pernambuco	Recife	PE	Brazil
Fabiana Costa Machado Zacharias	Universidade de São Paulo	São Paulo	SP	Brazil
Fabiana Martins Dias de Andrade	Universidade Federal de Minas Gerais	Belo Horizonte	MG	Brazil
Fabiana Morandi Jordão	Universidade Federal de Mato Grosso	Barra do Garças	MT	Brazil
Fernando Guimarães	Universidade Estadual de Campinas	Campinas	SP	Brazil
Flávia Reis de Andrade	Universidade de Brasília	Brasília	DF	Brazil
Flávia Tavares Silva Elias	Fundação Oswaldo Cruz	Brasília	DF	Brazil
Franciele Marabotti Costa Leite	Universidade Federal de Pelotas	Pelotas	RS	Brazil
Franciele Silvia Carlo	Universidade Federal de Mato Grosso	Cuiabá	MT	Brazil
Francieli Ferreira de Andrade Batista	Universidade Estadual de Londrina	Londrina	PR	Brazil
Francisco Edilson Ferreira de Lima Júnior	Ministério da Saúde	Brasília	DF	Brazil
Francisco Jose Dutra Souto	Universidade Federal de Mato Grosso	Mato Grosso	MT	Brazil
Francisco Roger Aguiar Cavalcante	Secretaria de Estado da Saúde do Ceará	Sobral	CE	Brazil
Frederico Alves Dias	Secretaria de Saúde do Estado do Paraná	Curitiba	PR	Brazil
Gabriela Callo	Universidade Federal de Pelotas	Pelotas	RS	Brazil
Gabriela de Cássia Ribeiro	Universidade Federal dos Vales do Jequitinhonha e Mucuri	Teófilo Otoni	MG	Brazil
Gabriela Tavares Magnabosco	Ministério da Saúde	Brasília	DF	Brazil
Gabriele Rissotto Menegazzo	Universidade Federal de Santa Maria	Santa Maria	RS	Brazil
Galba Freire Moita	Ministério da Saúde	Fortaleza	CE	Brazil
Glaciene Mary da Silva Gonçalves	Centro de Pesquisas Aggeu Magalhães	Recife	PE	Brazil
Glaucia Maria Bressan	Universidade Tecnológica Federal do Paraná	Cornélio Procópio	PR	Brazil
Guilherme Carneiro Reckziegel	Universidade de Brasília	Brasília	DF	Brazil
Gustavo Botelho Sampaio	Hospital da Criança e Maternidade	São José do Rio Preto	SP	Brazil
Gustavo Laine	Ministério da Saúde	Brasília	DF	Brazil
Gustavo Libério de Paulo	Pontifícia Universidade Católica de Minas Gerais	Belo Horizonte	MG	Brazil
Hayla Nunes da Conceição	Universidade Federal do Piauí	Teresina	PI	Brazil
Heloisa Nascimento	Universidade Federal do Oeste do Pará	Santarém	PA	Brazil
Henry Maia Peixoto	Universidade de Brasília	Brasília	DF	Brazil
Hernane Guimarães Santos	Universidade Federal do Oeste do Pará	Belém	PA	Brazil
Igor Gonçalves Ribeiro	Universidade Federal dO Tocantins	Palmas	TO	Brazil
Indianara Maria de Barros Canuto	Fundação Oswaldo Cruz	Recife	PE	Brazil
Ingrid Cristiane Pereira Gomes	Universidade Federal de Sergipe	Aracaju	SE	Brazil
Ione Ayala Gualandi de Oliveira	Fundação Oswaldo Cruz	Rio de Janeiro	RJ	Brazil
Isabel Cristina Martins Emmerick	Fundação Oswaldo Cruz	Rio de Janeiro	RJ	Brazil
Isabella Bagni	Universidade Estadual de Campinas	Campinas	SP	Brazil
Isis de Freitas Espeschit Braga	Universidade Federal de Juiz de Fora	Juiz de Fora	MG	Brazil
Ítala Mônica de Sales Santos	Universidade Federal do Ceará	Sobral	CE	Brazil
Italo Wesley Oliveira Aguiar	Universidade Federal do Ceará	Fortaleza	CE	Brazil
Ivone Evangelista Cabral	Universidade Federal do Rio de Janeiro	Rio de Janeiro	RJ	Brazil
Jacques Antonio Cavalcante Maciel	Universidade Federal do Ceará	Fortaleza	CE	Brazil
Jaluza Aimèe Schneider	Universidade do Vale do Rio dos Sinos	São Leopoldo	RS	Brazil
Janete Maria Rebelo Vieira	Fundação Oswaldo Cruz	Rio de Janeiro	RJ	Brazil
Jeane Glaucia Tomazelli	Instituto Nacional de Câncer	Rio de Janeiro	RJ	Brazil
Jefferson Paixão Cardoso	Universidade Estadual do Sudoeste da Bahia	Vitória da Conquista	BA	Brazil
Jefferson Pereira Caldas dos Santos	Universidade Federal do Piauí	Teresina	PI	Brazil
Jesem Douglas Yamall Orellana	Fundação Oswaldo Cruz	Manaus	AM	Brazil
João Paulo Cola	Universidade do Estado do Rio de Janeiro	Rio de Janeiro	RJ	Brazil
João Paulo Toledo	Organização Pan - americana da Saúde	Brasília	DF	Brazil
João Roberto Cavalcante	Universidade do Estado do Rio de Janeiro	Rio de Janeiro	RJ	Brazil
Jonas Lotufo Brant de Carvalho	Universidade de Brasília	Brasília	DF	Brazil
José Anael Neves	Universidade Federal da Paraíba	João Pessoa	PB	Brazil
José Fernando Andrade Costa	Universidade Estadual de Feira de Santana	Feira de Santana	BA	Brazil
José Leopoldo Ferreira Antunes	Universidade de São Paulo	São Paulo	SP	Brazil
Josivania Arrais de Figueiredo	Ministério da Saúde	Brasília	DF	Brazil
Joslaine de Oliveira Nunes	Ministério da Saúde	Brasília	DF	Brazil
Julia Hiromi Hori Okuyama	Universidade Federal de São Paulo	São Paulo	SP	Brazil
Juliana Balbinot Hilgert	Universidade Federal do Rio Grande do Sul	Porto Alegre	RS	Brazil
Jurema Correa da Mota	Fundação Oswaldo Cruz	Rio de Janeiro	RJ	Brazil
Kátia Fernanda Alves Moreira	Universidade Federal de Rondônia	Porto Velho	RO	Brazil
Kátia Jakovljevic Pudla Wagner	Universidade Federal de Santa Catarina	Curitibanos	SC	Brazil
Keitty Cordeiro de Andrade	Universidade de Brasília	Brasília	DF	Brazil
Kellyn Kessiene de Sousa Cavalcante	Universidade Federal do Ceará	Fortaleza	CE	Brazil
Kesia Tomasi da Rocha	Universidade Católica do Rio Grande do Sul	Porto Alegre	RS	Brazil
Klauss Kleydmann Sabino Garcia	Ministério da Saúde	Brasília	DF	Brazil
Laís Santos de Magalhães Cardoso	Universidade Federal de Minas Gerais	Belo Horizonte	MG	Brazil
Lara Livia Santos da Silva	Universidade Federal de Goiás	Goiânia	GO	Brazil
Larissa Zepka Baumgarten	Secretaria de Educação do Estado do Paraná	Curitiba	PR	Brazil
Lays Janaina Prazeres Marques	Universidade de São Paulo	São Paulo	SP	Brazil
Leonardo Pestillo de Oliveira	Universidade Cesumar	Maringá	PR	Brazil
Leonardo Ponte Marques	Universidade de Fortaleza	Fortaleza	CE	Brazil
Lia Borges de Mattos Custodio	Universidade Estadual Paulista	São Paulo	SP	Brazil
Liandro da Cruz Lindner	Universidade de São Paulo ‘’Júlio de Mesquita Filho’’	São Paulo	SP	Brazil
Lídia Bezerra Barbosa	Universidade Federal de Alagoas	Maceió	AL	Brazil
Lindsay Ximenes Fonseca	Universidade de Brasília	Brasília	DF	Brazil
Lilian Kelen de Aguiar	Universidade Federal de Minas Gerais	Belo Horizonte	MG	Brazil
Liliane Barbosa Amorim	Instituto Federal do Maranhão Campus Itapecuru-Mirim	Itapecuru-Mirim	MA	Brazil
Loren Queli Pereira	Universidade Federal do Triângulo Mineiro	Uberaba	MG	Brazil
Lucas Monteiro Santos	Universidade Federal da Bahia	Salvador	BA	Brazil
Luceime Olívia Nunes	Faculdade de Medicina de Botucatu	Botucatu	SP	Brazil
Lúcia Helena Linheira Bisetto	Universidade de São Paulo	São Paulo	SP	Brazil
Lúcia Yasuko Izumi Nichiata	Universidade de São Paulo	São Paulo	SP	Brazil
Luciana Santos Dubeux	Instituto de Medicina Integral Professor Fernando Figueira	Recife	PE	Brazil
Luciano Pamplona de Góes Cavalcanti	Universidade Estadual de Feira de Santana	Feira de Santana	BA	Brazil
Lucidio Clebeson de Oliveira	Universidade do Estado do Rio Grande do Norte	Açu	RN	Brazil
Luís Carlos Lopes-Júnior	Universidade Federal do Espírito Santo	Vitória	ES	Brazil
Luis Felipe Pupim dos Santos	Universidade Estadual Paulista ‘’Júlio de Mesquita Filho’’	São Paulo	SP	Brazil
Luiz Henrique Picolo Furlan	Universidade Federal do Paraná	Curitiba	PR	Brazil
Luiza Jane Eyre de Souza Vieira	Universidade de Fortaleza	Fortaleza	CE	Brazil
Lygia Carmen de Moraes Vanderlei	Instituto de Medicina Integral Professor Fernando Figueira	Recife	PE	Brazil
Mabel Miluska Suca Salas	Universidade Federal de Juiz de Fora	Juiz de Fora	MG	Brazil
Malvina Thais Pacheco Rodrigues	Universidade Federal do Piauí	Teresina	PI	Brazil
Marcelo Augusto Oliveira de Sales	Universidade Federal da Paraíba	João Pessoa	PB	Brazil
Marcio Denis Medeiros Mascarenhas	Universidade Federal do Piauí	Teresina	PI	Brazil
Marco Antônio Natal Vigilato	Organização Pan-Americana da Saúde	San Salvador	-	El Salvador
Marcos Antonio da Silva Cristovam	Universidade Paranaense	Umuarama	PR	Brazil
Marcos Venicius Malveira de Lima	Secretaria de Estado da Saúde do Acre	Rio Branco	AC	Brazil
Margarida Cristiana Napoleão Rocha	Universidade de Brasília	Brasília	DF	Brazil
Maria Auxiliadora Santos Soares	Secretaria Municipal da Saúde de Salvador	Salvador	BA	Brazil
Maria Catarina Salvador da Motta	Universidade Federal do Rio de Janeiro	Rio de Janeiro	RJ	Brazil
Maria Cristina Antunes Willemann	Faculdade de Ciências Médicas da Santa Casa de São Paulo	São Paulo	SP	Brazil
Maria da Consolação Magalhães	Universidade Federal de Juiz de Fora	Juiz de Fora	RJ	Brazil
Maria Dalva Barbosa Baker Méio	Fundação Oswaldo Cruz	Rio de Janeiro	RJ	Brazil
Maria Helena Ruzany	Universidade do Estado do Rio de Janeiro	Rio de Janeiro	RJ	Brazil
Maria Isabel Nascimento	Universidade Federal Fluminense	Rio de Janeiro	RJ	Brazil
Maria Laura Braccini Fagundes	Secretaria da Saúde do Rio Grande do Sul	Cachoeira do Sul	RS	Brazil
Maria Luiza Doria Almeida	Universidade Federal do Espírito Santo	Vitória	ES	Brazil
Maria Rosana Issberner Panachão	Instituto Adolfo Lutz	São Paulo	SP	Brazil
Maria Rosilene Cândido Moreira	Universidade Federal do Cariri	Juazeiro do Norte	CE	Brazil
Maria Yaná Guimarães Silva Freitas	Universidade Estadual de Feira de Santana	Feira de Santana	BA	Brazil
Mariana Mapelli de Paiva	Instituto Federal de Educação, Ciência e Tecnologia do Norte de Minas Gerais	Montes Claros	MG	Brazil
Mariana Silveira Echeverria	Universidade Federal de Pelotas	Pelotas	RS	Brazil
Mariana Sousa Lopes	Universidade Federal de Minas Gerais	Belo Horizonte	MG	Brazil
Mariane de Mello Fontanelli	Universidade de São Paulo	São Paulo	SP	Brazil
Mario Jorge Sobreira da Silva	Instituto Nacional de Câncer	Rio de Janeiro	RJ	Brazil
Martha Maria de Albuquerque Belo	Secretaria de Estado da Saúde da Paraíba	João Pessoa	PB	Brazil
Matheus de Sousa Mata	Universidade Federal do Rio Grande do Norte	Natal	RN	Brazil
Michele Souza e Souza	Fundação Oswaldo Cruz	Rio de Janeiro	RJ	Brazil
Micheline Mendes	Fundação Oswaldo Cruz	Recife	PE	Brazil
Naiza Nayla Bandeira de Sá	Universidade Federal do Pará	Pará	PA	Brazil
Natália Bastos Ferreira	Universidade Regional do Cariri	Cariri	CE	Brazil
Natan Monsores	Universidade de Brasília	Brasília	DF	Brazil
Nathália França de Oliveira	Universidade do Estado do Amazonas	Manaus	AM	Brazil
Nayara Figueiredo Vieira	Universidade Federal de Goiás	Goiânia	GO	Brazil
Nayore Tamie Takamiya	Universidade Federal de São Carlos	São Carlos	SP	Brazil
Neilane Bertoni	Instituto Nacional de Câncer	Rio de Janeiro	RJ	Brazil
Nídia Figueiredo Carneiro	Universidade Estadual de Montes Claros	Montes Claros	MG	Brazil
Olga Maíra Machado Rodrigues	Universidade de Brasília	Brasília	DF	Brazil
Oriana Deyze Correia Paiva Leadebal	Universidade Federal da Paraíba	João Pessoa	PB	Brazil
Orlando Luiz do Amaral Júnior	Universidade Federal de Santa Maria	Santa Maria	RS	Brazil
Osmar de Oliveira Cardoso	Universidade Federal do Piauí	Teresina	PI	Brazil
Pamela Alejandra Escalante Saavedra	Conselho Federal de Farmácia	Brasília	DF	Brazil
Pâmela Cristina Gaspar	Organização Pan-Americana de Saúde	Brasília	DF	Brazil
Pamila Siviero	Universidade Federal de Alfenas	Alfenas	MG	Brazil
Patrícia Barreto Cavalcanti	Universidade Federal da Paraíba	João Pessoa	PB	Brazil
Patricia Emanuella Ramos Marzola	Universidade Federal de Santa Catarina	Florianópolis	SC	Brazil
Patrícia Maria Oliveira Machado	Universidade Federal da Integração Latino-Americana	Foz do Iguaçu	PR	Brazil
Paula Martins Santucci	Faculdade de Saúde Pública	São Paulo	SP	Brazil
Pauline Lorena Kale	Universidade Federal do Rio de Janeiro	Rio de Janeiro	RJ	Brazil
Paulo Leonardo Ponte Marques	Universidade de Fortaleza	Fortaleza	CE	Brazil
Paulo Victor Cesar de Albuquerque	Universidade Federal de Pelotas	Pelotas	RS	Brazil
Priscila Carminati Siqueira	Universidade Federal do Espírito Santo	Vitória	ES	Brazil
Priscila Stacul	ABEU Centro Universitário	Belford Roxo	RJ	Brazil
Rafael Alves Guimarães	Universidade Federal de Goiás	Rio Verde	GO	Brazil
Rafaela Albuquerque	Universidade de Brasília	Brasília	DF	Brazil
Rafaela Almeida Schiavo	Instituto MaterOnline de Psicologia Perinatal	São Paulo	SP	Brazil
Raimundo Ferreira Lima	Centro Universitário Fametro	Fortaleza	CE	Brazil
Raphael Mendonça Guimarães	Fundação Oswaldo Cruz	Rio de Janeiro	RJ	Brazil
Rayane Cavalcante Pereira Batista	Universidade de Brasília	Brasília	DF	Brazil
Rebecca Faray Ferreira Lopes	Universidade do Estado do Rio de Janeiro	Rio de Janeiro	RJ	Brazil
Régia Maria Batista Leite	Universidade de Pernambuco	Garanhuns	PE	Brazil
Reinaldo Antonio Silva-Sobrinho	Universidade Estadual do Oeste do Paraná	Foz do Iguaçu	PR	Brazil
Renata Iani Werneck	Pontifícia Universidade Católica do Paraná	Curitiba	PR	Brazil
Ricardo Castanho Moreira	Universidade Estadual do Norte do Paraná	Jacarezinho	PR	Brazil
Ricardo Franklin de Freitas Mussi	Universidade do Estado da Bahia	Salvador	BA	Brazil
Rodrigo Pinheiro de Toledo Vianna	Universidade Federal da Paraíba	João Pessoa	PB	Brazil
Roger Keller Celeste	Universidade do Estado do Rio de Janeiro	Rio de Janeiro	RJ	Brazil
Rogério Lessa Horta	Universidade FEEVALE	Rio Grande	RS	Brazil
Rosália Garcia Neves	Universidade Federal de Pelotas	Pelotas	RS	Brazil
Rosemeire de Olanda Ferraz	Universidade Estadual de Campinas	Campinas	SP	Brazil
Rubia Laine de Paula Andrade	Universidade de São Paulo	São Paulo	SP	Brazil
Samia Abdul Samad	Organização Pan-Americana da Saúde	Brasília	DF	Brazil
Samia Samad	Organização Pan-americana da Saúde	Brasília	DF	Brazil
Sandra Costa Fonseca	Universidade Federal Fluminense	Niterói	RJ	Brazil
Sandra Valongueiro Alves	Universidade Federal de Pernambuco	Recife	PE	Brazil
Shaiana Vilella Hartwig	Universidade do Estado de Mato Grosso	Cuiabá	MT	Brazil
Silvia Cristina Martini Rodrigues	Universidade de Mogi das Cruzes	Mogi das Cruzes	SP	Brazil
Silvia Giselle Ibarra Ozcariz	Universidade Federal de Santa Catarina	Florianópolis	SC	Brazil
Sueli Fumie Yamada-Ogatta	Universidade Estadual de Londrina	Londrina	PR	Brazil
Suely Arruda Vidal	Instituto de Medicina Integral Professor Fernando Figueira	Recife	PE	Brazil
Suely Gandolfi Dallari	Universidade de São Paulo	São Paulo	SP	Brazil
Suzana Ferreira Paulino Domingos	Universidade Federal Rural de Pernambuco	Recife	PE	Brazil
Suzana Madeira Diório	Instituto Lauro de Souza Lima	Manaus	AM	Brazil
Tamara Leite Cortez	Prefeitura Municipal de São Paulo	São Paulo	SP	Brazil
Teresa Cristina Vieira Segatto	Universidade Federal do Rio Grande do Norte	Natal	RN	Brazil
Thaynã Ramos Flores	Universidade Federal de Pelotas	Pelotas	RS	Brazil
Thiago Dias Sarti	Universidade Federal do Espírito Santo	Espírito Santo	ES	Brazil
Thiago Nascimento do Prado	Universidade de Brasília	Brasília	DF	Brazil
Valter Duro Garcia	Complexo Hospitalar Irmandade Santa Casa de Misericórdia de Porto Alegre	Porto Alegre	RS	Brazil
Vanessa Canto	Universidade Federal de Santa Catarina	Florianópolis	SC	Brazil
Vânia Carvalho Santos	Universidade Federal de Sergipe	São Cristóvão	SE	Brazil
Vania dos Santos	Universidade de São Paulo	São Paulo	SP	Brazil
Virginia Junqueira	Universidade Federal de São João del-Rei	São João del-Rei	MG	Brazil
Virginia Kagure Wachira	Universidade de Brasília	Brasília	DF	Brazil
Vivian Siqueira Santos Gonçalves	Universidade de Brasília	Brasília	DF	Brazil
Wallace dos Santos	Secretaria do Estado de Saúde	Brasília	DF	Brazil
Waneska Alves	Universidade Federal de Juiz de Fora	Governador Valadares	MG	Brazil
Wanessa da Silva de Almeida	Fundação Oswaldo Cruz	Rio de Janeiro	RJ	Brazil
Washington Leite Junger	Universidade do Estado do Rio de Janeiro	Rio de Janeiro	RJ	Brazil
Wendel Mombaque dos Santos	Universidade de São Paulo	São Paulo	SP	Brazil
Willian Augusto de Melo	Universidade do Paraná	Paranavaí	PR	Brazil
Yves Mauro Fernandes Ternes	Secretaria Municipal de Saúde de Goiânia	Goiânia	GO	Brazil

